# Retrospective analysis of 131 feline uroliths from the Republic of Ireland and Northern Ireland (2010-2020)

**DOI:** 10.1186/s13620-023-00232-1

**Published:** 2023-02-06

**Authors:** Cristina J. Ortega, Evangelia M. Stavroulaki, Amanda Lawlor, Jody Lulich, Benoit Cuq

**Affiliations:** 1 Section of Small Animal Clinical Studies, University College Dublin School of Veterinary Medicine, Belfield, Dublin, Ireland; 2 Minnesota Urolith Center, Department of Veterinary Clinical Sciences, College of Veterinary Medicine, University Minnesota, St Paul, MN USA

**Keywords:** Urolith, Ireland, Cats, Struvite, Calcium oxalate

## Abstract

**Background:**

The proportions of different urolith types have not been investigated in cats from the Republic of Ireland (ROI) and Northern Ireland (NI) previously. The objective of this study was to investigate the proportions of different feline urolith types submitted to Minnesota Urolith Center from the ROI and NI from 2010 to 2020. An additional aim of this study was to identify potential risk factors associated with each urolith type in cats in this geographic area.

**Results:**

One hundred and thirty-one uroliths were submitted for the studied period with 44.3% being struvite, 43.5% calcium oxalate and 7.6% compound. Only 11 uroliths were submitted in the first 4 years. The number of submissions increased after 2015, peaking in 2019 with 25 submissions. Due to low numbers no conclusions could be made about changes in incidence of urolith types over time. Cats ≤7 years of age were significantly more likely to be diagnosed with struvite uroliths (OR, 2.87 [1.37-6.06]; *p* = 0.007) while cats ≥7 years of age with calcium oxalate uroliths (OR, 2.67, [1.29-5.37], *p* = 0.004).

**Conclusions:**

This is the first epidemiologic study of urolithiasis from cats in the ROI and NI. The most prevalent types of uroliths in our study population were struvite and calcium oxalate. Due to the low number of urolith submissions, changes in the incidence of different uroliths could not be accurately determined. Increasing age was associated with calcium oxalate formation while younger cats were more commonly diagnosed with struvite urolithiasis which can be medically dissolved. Therefore, urolith dissolution is more likely to be successful in young cats than older cats.

## Background

Urolithiasis is a common pathologic condition in dogs and cats and has been associated with significant morbidity and mortality in both species [1]. Stone formation has been associated with familial, congenital, or acquired pathophysiological factors that progressively increase the risk of precipitation of excretory metabolites in urine [2, 3]. Twelve to 22% of cats with lower urinary tract disease (LUTD) have been reported to have urolithiasis [4]. In a previous study, cats with urolithiasis and LUTD were hospitalized significantly more often than cats with LUTD such as sterile or bacterial cystitis [4]. Urinary tract obstruction from any cause can impair kidney function leading to acute kidney injury (AKI) [5]. In one study, the prevalence of chronic kidney disease (CKD) among cats with urolithiasis was significantly higher than that of patients without urolithiasis (56% vs 30%, respectively) [6]. Similar associations between CKD and urolithiasis have also been found in human patients [7–9].

Previous studies have demonstrated that the two most common types of uroliths in cats are calcium oxalate (CaOx) and magnesium ammonium phosphate (struvite) [1, 2, 10–13]. In cats, struvite was the most common type of urolith during the early 1980s, representing 78% of the uroliths submitted to the Minnesota Urolith Center (MUC) [1]. However, a change in urolith composition has been reported since then and, in 2002, CaOx represented 55% of feline uroliths submission to the MUC while only 33% were categorized as struvite [1]. More recently the proportion of struvite stones appears to be increasing, counting up to 47% in one study [2]. The proportion of CaOx-containing uroliths is reported to vary between 41 and 69% in recent retrospective studies carried out in the United States, Canada, and the Netherlands [2, 3, 10, 14]. Differences in the proportions of uroliths might exist among countries including the Republic of Ireland (ROI) and Northern Ireland (NI) given the relatively small breeding population and the differences in breed popularity.

Risk factors may differ according to the type of urolith, sex, breed, age, and neuter status [11, 15–19]. To the authors knowledge, an epidemiological study evaluating the prevalence of uroliths, and risk factors associated with urolith formation has not been performed in cats from Ireland but could lead to specific recommendations.

The purpose of this study was to describe the proportions of different urolith types and determine risk factors associated with each type of urolith in cats from the ROI and NI from 2010 to 2020.

## Methods

### Case selection

Electronic records of uroliths submitted to the MUC (College of Veterinary Medicine, University of Minnesota, St Paul, MN, USA) retrieved from cats from the ROI and NI between January 2010 and December 2020 were evaluated. For each urolith, data extracted included urolith composition and localization, and the signalment of patients diagnosed with urolithiasis and information about previous episodes of urolithiasis, concurrent diseases, and urine culture when available. Full medical records were retrieved and reviewed for uroliths submitted from the University College Dublin Veterinary Hospital (UCDVH).

### Urolith analysis

Urolith quantitative analysis was performed using polarizing light microscopy and/or infrared spectroscopy. Only the uroliths containing more than 70% of a biogenic mineral were classified as that mineral type [3]. A urolith without a nidus or shell but that contained ≥70% of a single mineral was identified by that mineral. A urolith without a nidus or shell that contained < 70% of any single mineral was referred to as a mixed urolith. Compound uroliths were defined as having a central core or outer layer containing ≥70% of a single mineral with an opposing outer layer or central core of a different mineral [20]. Less prevalent uroliths (calcium phosphate apatite, ammonium urate, mixed stones, and miscellaneous material) were classified as “Other” [20].

### Statistical analysis

Collected data were analyzed using commercially available statistical software packages (GraphPad Prism version 9.0 and SPSS version 23.0). Descriptive statistics included calculation of count and percentage for categorical variables while for continuous variables, the median and range were evaluated. Age was divided into two categories based on the median. Fisher’s exact test was used to test for associations between urolith type and categorical variables including gender, age, breed, and retrieval source. Odds ratios (OR) with 95% confidence intervals (CI) were also calculated using the Baptista-Pike method to analyze whether there is an association between breed, age, gender and urolith type. Statistical significance was set at *p* < 0.05.

## Results

**Fig. 1:**
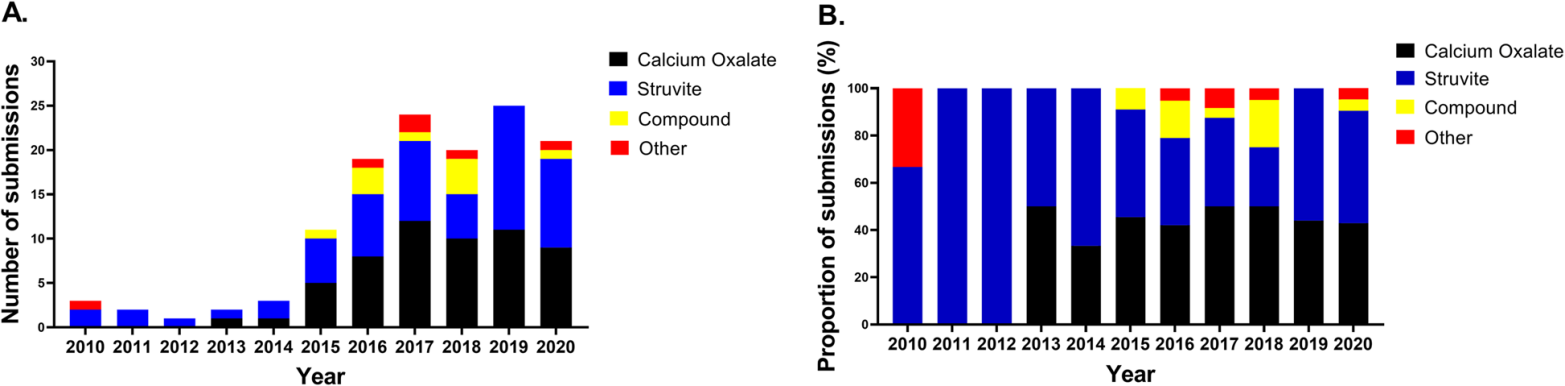
**A** Annual number of uroliths in cats from the ROI and NI submitted for analysis to MUC from 2010 to 2020. **B** Annual proportions of struvite-, calcium oxalate-, compound and other-containing uroliths in cats from the ROI and NI. Calcium phosphate apatite, ammonium urate, mixed stones, and miscellaneous material are classified as “Other”

A total of 131 uroliths submitted from cats in the ROI and NI were analyzed by the MUC between January 1, 2010, and December 31, 2020. Seventeen uroliths were submitted from a teaching veterinary referral hospital (UCD Veterinary Hospital) and 114 uroliths from 56 private practices (49 from the ROI and 7 from NI). During the 10-year study period, the annual submission rate of feline uroliths increased from 3 in 2010 to 21 in 2020, with a peak in 2019 of 25 submissions (Fig. 1). Given the low number of submissions, especially during the first 4 years of the study (a total of 11 submissions), no conclusions could be made regarding trends in urolith proportions from 2010 to 2020 in ROI and NI.

### Patient population

Patient characteristics including breed, age, gender, and neutered/castration status are listed in Table 1. Medical history was available for 76/131 (58%) submissions with no significant concurrent disease reported on 63/76 (48%) submissions. Four of the 13 cats with reported concomitant diseases were diagnosed with chronic kidney disease, and one cat was diagnosed with idiopathic hypercalcaemia. Twelve cats were reported to have at least one previous episode of urolithiasis. Culture results were available from 18 cats with 16 being negative (90%). *Escherichia coli* was isolated in a cat that had struvite uroliths and multiple microorganisms were isolated from the urine of another cat that had calcium oxalate stones.

**Table 1:** Individual data associated with 131 feline uroliths submitted to the Minnesota Urolith Center

Subcategory	Portion	Percentage
**Gender**		
Female entire	24/128	18.75%
Female neutered	37/128	28.91%
Male entire	25/128	19.53%
Male neutered	42/128	32.81%
**Age**		
≤ 7 years	81/128	63.28%
> 7 Years	47/128	36.72%
**Breed**		
Domestic Shorthair	85/125	68.00%
European Shorthair	14/125	11.20%
Domestic longhair	8/125	6.40%
Persian	4/125	3.20%
Other	14/125	11.20%
**Retrieval method**		
Surgical	99/118	83.89%
Voided	11/118	9.32%
Catheter	7/118	5.93%
Laparoscopic-assisted cystotomy	1/118	0.85%
**Stone location**		
Kidney	4/128	3.13%
Ureter	5/128	3.91%
Bladder	106/128	82.81%
Urethra	14/128	10.94%

### Urolith location

The majority of uroliths were isolated from the lower urinary tract (120/128, 93.8%) while the rest were isolated from the ureter (5/128, 3.9%) and/or the kidney (4/128, 3.1%) (Table 1).

### Urolith types and risk factors

Of the 131 submissions, 58 uroliths were classified as struvite (44.3%), 57 were classified as calcium oxalate (43.5%), 10 as compound uroliths (7.6%), and the remaining 6 were classified as “Others” (Fig. 1).

Cats 7- year-old or younger were significantly more likely to be diagnosed with struvite uroliths (OR, 2.87 [1.37-6.06]; *p* = 0.007) while cats older than 7 years were more likely to have calcium oxalate uroliths (OR, 2.67, [1.29-5.37], *p* = 0.004; Table 2). No other significant associations were found between gender, neuter status or urolith location.

**Table 2:** Age groups and stone composition association in cats from the ROI and NI expressed as odds ratios (OR) with 95% confidence intervals. Statistical significance was set at *p* < 0.05

Age	Stone composition		*p*-value	OR	95% CI
Age ≤ 7 years	Struvite (*n* = 40/81)	Non-struvite (*n* = 31/81)	0.007	2.87	1.37-6.06
Age > 7 years	CaOx (24/47)	Non-CaOx (23/47)	0.012	2.67	1.29-5.37

## Discussion

The purpose of this study was to describe the proportions of different urolith types in cats from the ROI and NI from 2010 to 2020 and identify potential risk factors associated with each urolith type. The number of urolith submissions over the study period has substantially increased. The most prevalent uroliths in cats from our study were struvite and CaOx. Younger cats were more likely to present struvite uroliths while older cats were more likely to be diagnosed with calcium oxalate uroliths. No other significant associations were found between gender, neuter status or urolith location.

In agreement with previous epidemiologic studies, struvite and CaOx uroliths were the most prevalent uroliths in cats, overall representing 87.8% of the urolith submissions in the present study [1, 2, 10–13]. Unfortunately, sample size was too small to detect any changes in proportions of urolith composition over the study period. In addition, in our study, only 7% of the uroliths were isolated from the upper urinary tract. It has been previously recognised that approximately 90% of the upper urinary tract uroliths are classified as CaOx [21]. Therefore, the true prevalence of CaOx urolithiasis in our study population has likely been underestimated as upper urinary tract uroliths were less commonly submitted. The true prevalence of struvite uroliths may also not coincide with the prevalence observed in this study since a dietary trial commonly precedes surgical treatment, potentially leading to their dissolution [22]. Urocystoliths not associated with bacteriuria usually resolve in 2-5 weeks with an appropriate diet change using a dissolution diet; avoiding the anesthetic and potential surgical complications of a cystotomy [22]. For this reason, there may be a significant number of struvite uroliths that have not been submitted for analysis following successful dissolution. Therefore, a prospective study with direct access to the medical history of each case would be necessary to analyze more accurately the prevalence of different types of uroliths.

It has been previously recognized that age is a predisposing factor for the development of certain types of uroliths. The data presented showed that cats older than 7 years of age were 2.7 times more likely to be diagnosed with CaOx uroliths. This is in agreement with previous studies [2, 19]. Calcium oxalate urolith formation results from the presence of calcium and oxalate in the urine [11]. Hypercalciuria can have various etiologies including hypercalcaemia (increased intestinal absorption, increased bone mobilisation) or increased renal excretion [11]. The most common diseases that have been associated with hypercalcemia in cats include malignancies, CKD, and idiopathic hypercalcemia [23, 24]. In our study, 55/131 submissions did not provide any medical history, while in 63/131 submissions, cats were classified as having a non-significant diseases; therefore, due to the retrospective nature of the study, there are not enough or strong data to confirm these hypotheses. Four cats were reported to have CKD and one cat was diagnosed with idiopathic hypercalcemia with all of them having calcium oxalate stones. It was previously demonstrated that cats with urolithiasis were predisposed to have CKD, and it is likely that this information might have been overlooked or omitted by referring veterinarians in the submission forms [6]. Moreover, the association with stage 1 CKD was not demonstrated in that retrospective study and such cats would likely be in the non-significant disease or even have no medical history provided [6]. Alternatively, it was previously reported that older cats have a significantly lower urine pH and a higher potential to form CaOx crystals compared to younger cats [10]. However, a recent study showed that the risk of CaOx was not increased only with changes in urine pH [25].

Comparatively, younger cats were 2.9 times more likely to have struvite uroliths. Struvite urolith formation results from the oversaturation of urine from magnesium, ammonium, and phosphate ions. In dogs, struvite urolithiasis more likely occurs due to a concurrent bacterial cystitis caused by urease-producing bacteria. In cats diagnosed with struvite urolithiasis, as in the present study, the urine is usually sterile [26]. However, in most cases of our study, previous antibiotic history was unknown, therefore no conclusions can be drawn from this data. A combination of dietary and possibly genetic factors has been associated with the formation of struvite uroliths in cats. Risk factors for the formation of struvite uroliths include increased dietary magnesium levels and/or an alkaline urine in combination of reduced water intake. Consuming a few large portions of food per day can lead to alkaline urine. This factor combined with low water intake may increase the risk of urolith and struvite crystal formation [26].

Similar to previously published data [2], bacteriuria was not commonly associated with urolithiasis in our cohort (11% of submitted urine cultures were positive) although the number of urine culture results was limited (18/131) and 42% (8/18) of cats from which urine culture results were available, had been previously treated with antibiotics. Only 2/18 samples cultured positive and *E. coli* was isolated in one of these. Given the limited information we have about the urine cultures performed, it is difficult to draw definitive conclusions regarding the relationship between bacteriuria and the development of uroliths in cats.

Among other risk factors investigated in our study, no significant gender or breed predisposition were reported to be associated with a particular urolith types. Conflicting results exist regarding gender and different urolith types with some studies reporting a higher frequency of CaOx uroliths in male cats and struvite uroliths in female cats and other studies not reporting any associations [2, 10, 13, 14]. CaOx formation has also been found to occur more frequently in purebred long-hair cats [11]. The only longhaired breed in this study was the Persian and they represented only 3% (4/125) of the data population; they were diagnosed with an equal frequency for struvite or CaOx uroliths. However, the small sample size in the present cohort might explain the lack of differences in breed or age predisposition for each individual uroliths.

Finally, it was very interesting to see an increase in the number of submissions in the second half of the study period: a total of 11 uroliths were submitted from 2010 to 2014 and this was followed by an average submission of 20 uroliths per year from 2015 to 2020. It is unlikely that such growth in submissions corresponds to a genuine increase in the incidence of uroliths as the number of uroliths submitted remains limited and the overall prevalence of urolithiasis in cats from Ireland is unknown. Despite the free service of MUC, it is possible that veterinarians were sending uroliths to different laboratories. Alternatively, awareness from practitioners in the ROI and NI must have increased to account for the greater urolith submission with improved communication from pet-food companies or continuing education. It is the authors’ belief that there has been an increase in the ownership of pet cats over the last 10 years with more owners seeking veterinary attention for these pets and this could have resulted in increased detection and treatment of urolithiasis. By conducting this retrospective descriptive study, the authors aimed to continue increasing awareness of the importance of submitting all uroliths for identification among veterinary practitioners in order to improve prevention and management recommendation in our geographic area.

Our study had several limitations due to its retrospective nature and the number of cats included. Unfortunately, the information provided by the veterinarians at the time of urolith submission was often incomplete or scarce. Only stones that were surgically removed, voided, or retrieved either with a catheter or minimally invasively were included. With newer surgical techniques (e.g., subcutaneous ureteral bypass) ureterotomy and nephrotomy are less commonly performed, thus it is likely that the proportion of uroliths removed from the upper urinary tract has decreased. Based on previous studies, it is therefore very likely that the actual prevalence of calcium oxalate uroliths would be higher. Lastly, data were derived only from submissions to the MUC from the ROI and NI; however other laboratories may be accessible to practitioners such as the Canadian Veterinary Uroliths Center or the UC Davis Gerald V. Ling Urinary Stone Analysis Laboratory [2, 3]. Therefore, our data might not be fully representative of all urolith identified in our geographical area, or cat’s signalment characteristics from other geographical areas.

## Conclusions

In this retrospective study, the most prevalent type of uroliths were struvite and calcium oxalate stones. Older cats were more commonly diagnosed with CaOx uroliths while younger cats presented struvite uroliths. The number of submissions from 2010 to 2020 has grown significantly. No gender, and neutering status associations were found with different urolith types in cats. However, this may be due to the small sample size.

Based on our results, some recommendations can be made to veterinarians practicing in Ireland. Considering the predisposition of young cats to develop struvite stones, in the presence of a non-obstructive radiopaque urolith in a young, otherwise healthy cat would be an indication for a dissolution trial prior to surgery given the likelihood of struvite urolithiasis. In addition, for older cats, surgical removal might be considered earlier, especially in the presence of findings from the history or biochemistry that suggest further CaOx urolithiasis. Completing the urolith submissions with a detailed patient and dietary history will help future epidemiological studies to thoroughly investigate risk factors associated with urolith formation. Future studies including a larger cohort of cats or prospective studies will be necessary to trend the changes in urolith proportions and potential predisposing factors.

## Data Availability

The datasets used and/or analyzed during the current study are available from the corresponding author on reasonable request.
